# Natural History of Degenerative Hip Abductor Tendon Lesions

**DOI:** 10.1177/03635465221135759

**Published:** 2022-11-22

**Authors:** Pascal Schenk, Dimitris Dimitriou, Stefan Rahm, Stefan M. Zimmermann, Michael Finsterwald, Kai Higashigaito, Reto Sutter, Patrick O. Zingg

**Affiliations:** * Department of Orthopedics, University Hospital Balgrist, University of Zürich, Zürich, Switzerland; ‡ Department of Radiology, University Hospital Balgrist, University of Zürich, Zürich, Switzerland; Investigation performed at the Department of Orthopedics, University Hospital Balgrist, University of Zürich, Zürich, Switzerland

**Keywords:** greater trochanteric pain syndrome (GTPS), hip abductor tendon tear, fatty infiltration, trochanteric bursitis, natural history

## Abstract

**Background:**

The best treatment of degenerative hip abductor tendon lesions remains largely unknown, as the natural course of the disease has not yet been reported. The aim of the present study was to investigate the natural history of symptomatic degenerative hip abductor lesions.

**Hypothesis:**

Nonoperatively treated hip abductor lesions progress over time, resulting in refractory hip pain and low functional outcomes.

**Study Design:**

Case series (prognosis); Level of evidence, 4.

**Methods:**

Consecutive patients with greater trochanteric pain syndrome and degenerative changes on magnetic resonance imaging (MRI) of the symptomatic hip were included. Bilateral hip MRI scans and a clinical examination were performed at a minimum follow-up of 36 months to study the type and location of hip abductor lesion. Progression of a lesion was defined as a more severe lesion as compared with the initial MRI results or if the lesion extended to another, initially not involved, trochanteric facet. The muscle’s fatty infiltration (FI) was also described.

**Results:**

From 106 patients identified, 58 patients (64 hips) aged 66 ± 14 years (mean ± SD) agreed to return to the clinic for follow-up MRI and met the inclusion criteria. At a mean 71-month follow-up, an overall 34% (22/64) of lesions had progressed over time: from trochanteric bursitis to tendinopathy (9/64, 14%) or partial tear (5/64, 8%), from tendinopathy to partial tear (4/64, 6%), from a partial to complete tear (3/64, 4.5%), and with 1 complete tear (1/64, 1.5%) extending to another trochanteric facet. Interestingly, 90% of partial tears remained stable or transformed into a scar. Although patients with a progressive lesion experienced more trochanteric pain (visual analog scale, 4.6 vs 2.8; *P* = .001), the functional outcomes were comparable with patients with a stable lesion. The majority of hips with a partial tear (64%) demonstrated a progression of gluteus minimus FI from a median grade of 1 to 2, whereas only 1 hip (3%) progressed from grade 2 to 3. Only 3 hips (9%) with a partial tear had a progression of gluteus medius FI, which did not differ significantly from the contralateral unaffected side.

**Conclusion:**

Nonoperative treatment might be a valid long-term option for degenerative hip abductor lesions, especially for partial tears, which demonstrated a low risk of clinically relevant progression or muscle FI and similar clinical outcomes to those reported in the literature for operatively treated hip abductor tendon lesions.

Greater trochanteric pain syndrome (GTPS) is a relatively common and debilitating condition with an annual incidence of 1.8 to 5.6 per 1000, and is commonly found in female patients between the ages of 40 and 60 years.^21,23,32^ The pathophysiology of GTPS remains unknown, although hip abductor tendinopathy is a frequent finding.^2,12^ Over the past decades, lesions of the gluteus medius (G*med*) and gluteus minimus (G*min*) tendons, the main hip abductors of the hip, have been identified as major causes of GTPS.^19,22^ The prevalence of abductor tears is estimated at 25% in patients undergoing a total hip arthroplasty for hip osteoarthritis^16^ and often causes insidious lateral hip pain without a history of acute trauma.^22^

Nonoperative treatment is one management option for degenerative hip abductor tears and consists of lifestyle modifications, weight loss, nonsteroidal anti-inflammatory drugs, targeted physical therapy,^36^ local corticosteroid injections,^20^ platelet-rich plasma injections,^25^ and shockwave therapy.^30^ Nonoperative treatment effectively relieves symptoms for most patients with GTPS,^14^ ranging from 48% to 80% depending on the treatment modality used (corticosteroid injections, shockwave therapy, and targeted physical therapy).^31^ However, if nonoperative treatment fails to provide pain relief and restore function after a minimum 3-month period^18^ or if G*med* or G*min* tendinitis and/or a partial tear is diagnosed on magnetic resonance (MR) imaging (MRI), surgical repair is usually recommended early to avoid chronic atrophy and fatty infiltration (FI) of the muscles.^17^ As a result, the natural course of nonoperatively treated hip abductor lesions shown on sequential MRI scans, to the best of our knowledge, has never been reported. Therefore, the aim of the present study was to investigate the natural history of symptomatic degenerative hip abductor lesions, specifically investigating clinical outcomes and lesion progression on MRI. We hypothesized that nonoperatively treated hip abductor lesions would progress over time, resulting in refractory hip pain and low functional outcomes.

## Methods

### Study Design and Inclusion and Exclusion Criteria

The present study was approved by the state ethical committee (BASEC 2018-01354) and was entirely conducted at our institution. Consecutive patients were identified who had GTPS, visited the outpatient clinic between January 2003 and November 2015, and underwent MRI of the symptomatic hip, which showed degenerative changes of the hip abductors. The diagnosis of GTPS was based on patient history (lateral hip pain localized to the greater trochanter, which is worse with weightbearing activities and side lying at night) and clinical examination (tenderness over the greater trochanter), whereas a pelvic radiograph was performed to exclude hip osteoarthritis.^12^ During that period, no patients with surgically treated GTPS were identified from our institutional database. MRI of the symptomatic hip was performed if patients had persistent symptoms for ≥6 months despite physical therapy and infiltration in the trochanteric bursa with cortisone and local anesthetic. Inclusion criteria were age >18 years with no history of surgery of the affected hip. Exclusion criteria were contraindication for MRI (eg, pacemaker or claustrophobia), severe hip osteoarthritis (Tönnis grade ≥3),^4^ hip dysplasia, any history of hip fracture or infection, inflammatory arthritis, and neurologic or musculoskeletal disorders, which could affect function, muscle tone, or degeneration. Patients who met the aforementioned criteria were invited to the outpatient clinic for a clinical examination and bilateral hip MRI. The minimum follow-up was 36 months.

### Radiographic Measurements

A standardized pelvic anteroposterior radiograph was performed at the initial presentation and at the last follow-up to measure the centrum-collum-diaphyseal (CCD) angle and femoral offset (horizontal distance from the femoral head center to the anatomic axis of the femur), which are commonly reported radiographic parameters associated with GTPS,^11^ as well as hip osteoarthritis according to Tönnis classification.^4^

### MR Examination

The MR images were acquired with a 3T Skyra-fit scanner (Siemens). Unilateral MRI was separately performed for each hip with the following sequences:

Coronal T2-weighted: repetition time/echo time, 4000 ms/83 ms; field of view, 220 mm; slice thickness, 4 mm
Sagittal T1-weighted: repetition time/echo time, 678 ms/14 ms; field of view, 180 mm; slice thickness, 4 mm
Transverse short tau inversion recovery: repetition time/echo time, 5000 ms/62 ms; inversion time, 210 ms; field of view, 180 mm; slice thickness, 7 mm
Transverse T1-weighted: repetition time/echo time, 820 ms/13 ms; field of view, 180 mm; slice thickness, 6 mm

**Figure 1.**
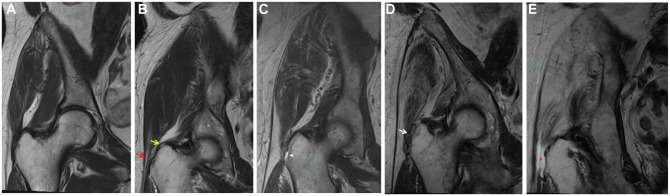
Coronal T2-weighted magnetic resonance images of a right hip demonstrating the types of hip abductor lesions classified from less to more severe: (A) intact tendon, (B) tendinopathy G*min* (upper arrow) and G*med* (lower arrow), (C) partial tendon tear of G*med* (arrowhead), (D) scar of G*med* and G*min* (arrow), (E) complete tendon tear of G*med* and G*min* (asterisk). G*med*, gluteus medius; G*min*, gluteus minimus.

The type of hip abductor lesion was defined from less to more severe as follows:

Trochanteric bursitis with intact tendons: fluid-filled bursa lateral to trochanter with hyperintensity in T2-weighted image and hypointensity on T1-weighted image (Figure 1A)
Tendinopathy: increased thickness and signal intensity of the tendon, especially on proton density fat-saturated images (Figure 1B)
Partial tendon tear: presence of fluid signals interrupting the fibers of the tendon (Figure 1C)
Scar: complete disruption on T1-weighted images, accompanied by a hypointense signal on proton density fat-saturated images (Figure 1D)
Complete tendon tear: complete disruption on T1-weighted images, accompanied by a marked hyperintense signal on proton density fat-saturated images (Figure 1E)

In the case of multiple tendon lesions (eg, anterior tendinopathy and lateral partial tear), only the most severe lesion was described.

**Figure 2.**
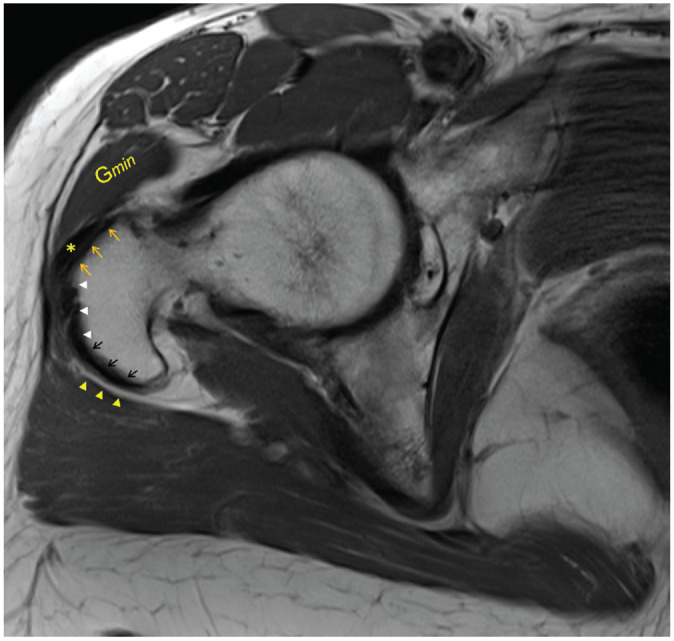
Axial T1-weighted magnetic resonance image of a right hip with intact hip abductors demonstrating the anterior (upper arrows), lateral (arrowheads), and posterior (lower arrows) facets. The gluteus minimus tendon (asterisk) is attached to the anterior facet and lies adjacent to the anterior part of the gluteus minimus muscle (G*min*). The trochanteric bursa (arrowheads opposite the black arrows) is also shown.

The location of the lesion was presented as anterior, lateral, posterior, or a combination of those, according to which trochanteric facet was involved, as described by Pfirrmann et al^28^ (Figure 2).

**Figure 3.**
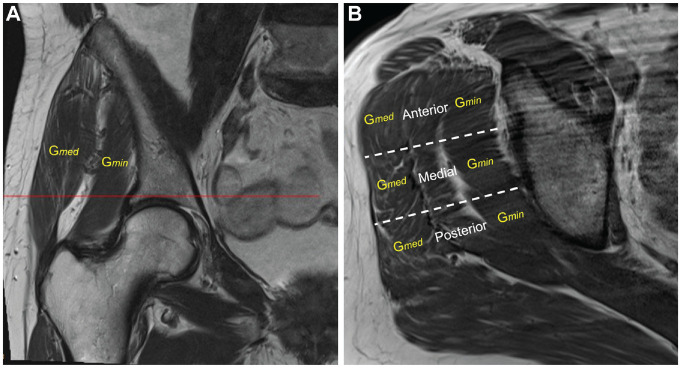
(A) Coronal T1-weighted magnetic resonance image of a right hip demonstrating the level where the muscle fatty infiltration (FI) was assessed (line). (B) Transverse T1-weighted magnetic resonance image of a right hip with intact hip abductors at the level of the acetabular roof showing the gluteus medius (G*med*) and gluteus minimus (G*min*) muscle. The FI of G*med* and G*min* was assessed at the anterior, medial, and posterior thirds and the median value estimated. The G*min* demonstrated a median grade of 0 FI, whereas the G*med* grade was 1.

The FI of the hip abductors was determined on the T1-weighted non–fat suppressed axial slice (owing to the improved definition of fatty tissue) at the level of the acetabulum roof (Figure 3A). A modified Goutallier/Fuchs^13^ classification was applied to describe the grade of FI: grade 0, normal muscle tissue; grade 1, fatty streaks; grade 2, FI but still more muscle than fat; grade 3, equal amounts of fat and muscle; grade 4, more fat than muscle.^3^ The FI of G*med* und G*min* was measured at the anterior, lateral, and posterior thirds (Figure 3B). The median FI was calculated for each muscle.

Progression of a lesion was defined as a more severe lesion as compared with the initial MRI description (eg, tendinopathy to partial tear) or if a lesion extended to another trochanter facet that was initially not involved. Stable lesions were defined as lesions that did not increase in severity and did not extend to other, initially not involved facets; a transformation from a partial lesion to a scar was also considered a stable lesion. All parameters of interest—hip osteoarthritis grade, type and location of the hip abductor lesion, FI of hip abductors—were assessed by 2 fellowship-trained musculoskeletal radiologists (R.S., K.H.) blinded to the patient’s clinical details. The radiologists performed the measurements individually and concurred with the results.

### Clinical Examination and Functional Outcomes

Chart review included pain, body mass index (BMI), and previous treatment plan (ie, physical therapy, infiltrations, shockwave therapy). The clinical examination consisted of the range of motion of the hip, muscle strength of the hip abductors in the lateral decubitus position, and the presence of limping. The Harris Hip Score (HHS)^35^ of the involved and contralateral sides as well as the Western Ontario and McMaster Universities Osteoarthritis Index (WOMAC),^37^ which is divided into 3 subscales consisting of pain (5 items), stiffness (2 items), and physical function (17 items), were acquired at the last follow-up. Pain assessment in the greater trochanter region was performed with a graphical visual analog scale (VAS)^26^ ranging from 0 to 10 points. The presence of a Trendelenburg gait was also recorded.

### Statistical Analysis

Descriptive statistics used mean, median, standard deviation, range, interquartile range, and percentages to present the data. All parameters were tested with the Kolmogorov-Smirnov test for normality. When the criteria for normality were met, a 2-tailed *t* test was used; otherwise, the Mann-Whitney test was applied to compare the different parameters between progressed and stable lesions. The Wilcoxon signed-rank test was applied to compare the muscle FI between initial presentation and last follow-up and between the ipsilateral and contralateral/unaffected hips at the last follow-up. A multiple logistic regression analysis was performed to evaluate whether lesion progression was correlated with patient demographics (age, BMI, smoking status), radiologic parameters (CCD angle, femoral offset, osteoarthritis grade), type, and location of the lesion. The level of significance was set to *P*≤ .05. All statistical analyses were performed using SPSS software (Version 23; IBM).

## Results

### Patient Characteristics and Radiographic Parameters

A total of 106 patients met the aforementioned inclusion criteria and were invited to the outpatient clinic for a clinical examination and bilateral hip MRI. Of that group, 58 patients (14 men, 44 women; 64 hips) aged 66 ± 14 years (mean ± SD; range, 33-91 years) agreed to return to the clinic for follow-up MRI (Table 1). The minimum follow-up for the 58 patients was 36 months.

**Table 1:** Patient Characteristics, Radiologic Parameters, Functional Scores, and Follow-up (64 Hips)^*a*^

Characteristic	Mean ± SD (Range) or No. (%)
Age, y	66 ± 14 (33-91)
Female sex	44 (69)
Right hip	38 (59)
BMI	26.7 ± 5 (19.2-35.8)
Smoking	28 (44)
Osteoarthritis: Tönnis classification	
Grade 0	23 (36)
Grade 1	37 (58)
Grade 2	4 (6)
CCD angle,^*b*^ deg	129 ± 6 (115-142)
Femoral offset, mm	50 ± 7 (36-66)
HHS,^*b*^ points	
Affected side	80 ± 15 (30-100)
Contralateral/unaffected side^*c*^	90 ± 14 (64-100)
WOMAC,^*b*^ points	2.5 ± 1.9 (0-6.9)
Follow-up, mo	71 ± 30 (38-194)

a BMI, body mass index; CCD, centrum-collum-diaphyseal; HHS, Harris Hip Score; WOMAC, Western Ontario and McMaster Universities Osteoarthritis Index.

b As measured at the final follow-up.

c A significant difference in HHS between affected and contralateral/unaffected sides at the last follow-up.

The average CCD angle was 129°± 6° and femoral offset 50 ± 7 mm. The majority of the patients (64%) also had mild hip osteoarthritis (Tönnis grade 1 or 2). A large proportion of patients (44%) were smoking during the follow-up period. The affected hip had a significantly lower HHS (80 ± 15 points) as compared with the contralateral unaffected hip (90 ± 14 points).

### Initial MR Examination

At the initial MR examination, 49 hips (77%) demonstrated a lesion of the hip abductors other than trochanteric bursitis (Table 2). The majority were partial tears (32 hips; 65%) involving mostly the anterior or lateral trochanteric facet (26 hips; 53% of all lesions), followed by tendinopathy (13 hips; 27%) involving mostly the anterior or lateral facet (10 hips; 20% of all lesions). A complete tear was observed in 3 hips (6%), each at the anterolateral facet.

**Table 2:** Hip Abductor Tendon Lesions at the Initial Presentation by Type and Location (49 Lesions)^*a*^

	Type of Lesion, No. (%)				
Location of Lesion	Tendinopathy	Partial Tear	Scar	Complete Tear	Total
Anterior	3 (6)	6 (12)			9 (18)
Lateral	2 (4)	7 (14)			9 (18)
Posterior		1 (2)			1 (2)
Anterolateral	4 (8)	12 (24)		3 (6)	19 (38)
Posterolateral	1 (2)	1 (2)			2 (4)
Whole tendon	3 (6)	5 (10)	1 (2)		9 (18)
Total	13 (26)	32 (64)	1 (2)	3 (6)	49 (100)

a Hips with isolated trochanteric bursitis (n = 15) without a lesion of the abductor tendon were excluded from the current analysis.

At the initial MR examination, 49 hips (77%) demonstrated a lesion of the hip abductors, and 15 hips (23%) had isolated trochanteric bursitis (Table 2). At the final follow-up, 56 hips (88%) had a hip abductor lesion, and 8 hips (12%) had isolated trochanteric bursitis (Table 3). Thus, 7 of the 15 hips that had only bursitis on the initial MRI developed abductor tears by the time of follow-up.

**Table 3:** Abductor Mechanism Lesions at the Final Follow-up by Type and Location (56 Lesions)^*a*^

	Type of Lesion, No. (%)				
Location of Lesion	Tendinopathy	Partial Tear	Scar	Complete Tear	Total
Anterior	2 (4)	5 (3)			7 (7)
Lateral	2 (4)	6 (11)	7 (13)		15 (28)
Posterior		1 (2)			1 (2)
Anterolateral	3 (5)	7 (13)	6 (11)	3 (5)	19 (33)
Posterolateral	5 (8)	1 (2)	1 (2)		7 (12)
Whole tendon	2 (4)	3 (5)	3 (5)	1 (2)	9 (16)
Total	14 (25)	21 (38)	17 (30)	4 (7)	56 (100)

a Hips with isolated trochanteric bursitis (n = 8) without a lesion of the abductor tendons were excluded from the analysis.

### MR Examination at the Last Follow-up

At the final follow-up, 56 hips (88%) demonstrated a hip abductor lesion other than trochanteric bursitis (Table 3). The majority were partial tears (21 hips; 38%) involving the anterior or lateral trochanteric facet (19 hips; 29% of all lesions), followed by scars (17 hips; 30%) involving mostly the anterior or lateral facet (14 hips; 25% of all lesions). A complete tear was observed in 4 hips (7%): 3 (5%) at the anterolateral facet and 1 (2%) involving the whole tendon.

### Lesion Progression

At an average 71-month follow-up, 34% of the lesions (22 hips) progressed from trochanteric bursitis to tendinopathy (9 hips; 14%) or partial tear (5 hips; 8%), from tendinopathy to partial tear (4 hips; 6%), and from a partial to a complete tear (3 hips; 4.7%), with 1 complete tear (1.5%) extending to another trochanteric facet. Interestingly, 90% of partial tears (26/29 hips) remained stable or transformed into a scar (Table 4).

**Table 4:** Lesion Progression as Reported at Final Follow-up (64 Lesions)

	Final Type of Lesion, No. (%)					
Initial Type of Lesion	No Lesion / Bursitis	Tendinopathy	Partial Tear	Scar	Complete Tear	Total
Bursitis	8 (12.5)	9 (14)^*a*^	5 (8)^*a*^			22 (34.5)
Tendinopathy		5 (8)	4 (6)^*a*^	1 (1.5)		10 (15.5)
Partial tear			12 (19)	13 (20)	3 (4.5)^*a*^	28 (44)
Scar				1 (1.5)		1 (1.5)
Complete tear				1 (1.5)	2 (3)^*b*^	3 (4.5)
Total	8 (12.5)	14 (22)	21 (33)	16 (25)	5 (7.5)	64 (100)

a Lesion progression.

b One of the 2 complete lesions progressed to another trochanter facet.

### Muscle FI

At the last follow-up, 35 hips (55%) demonstrated a progression of G*min* FI; 7 hips (11%), a progression of G*med* FI; and 4 hips (6%), a progression of G*min* and G*med* FI (Table 5). The G*min* FI in patients with a partial tear differed significantly (grade 2) from the contralateral unaffected hip (grade 1). The majority of the G*min* FI was grade 1, which progressed to grade 2, whereas only 1 hip (3%) progressed from grade 2 to 3 at the last follow-up. Just 3 hips (9%) demonstrated a progression of G*med* FI in hips with a partial tear, but no significant difference was observed as compared with the contralateral/unaffected side.

**Table 5:** Muscle Fatty Infiltration at Initial Presentation and Final Follow-up^*a*^

	FI (Goutallier Grade), Median							
	Initial Ipsilateral		Last Ipsilateral		Progression, No. (%)		Last Contralateral	
Initial Type of Lesion	G*min*	G*med*	G*min*	G*med*	G*min*	G*med*	G*min*	G*med*
Bursitis (n = 15)	1	1	1.25^*b*^	1	6 (40)	3 (20)	1	1
Tendinopathy (n = 13)	1	1	1.5^*b*^	1	6 (46)	1 (8)	2	1
Partial tear (n = 32)	1	1	2^*b*^	1	22 (69)	3 (9)	1^*c*^	1
Scar (n = 1)	2	1	2	2			2	2
Complete tear (n = 3)	4	1	4	1	1 (33)		4	1
Total (N = 64)	1	1	2^*b*^	1	35 (64)	7 (11)	1	1

a FI, fatty infiltration; G*med*, gluteus medius; G*min*, gluteus minimus.

b A significant difference between initial and last ipsilateral muscle FI.

c A significant difference between ipsilateral and contralateral muscle FI at last follow-up.

### Subgroup Analysis Between Patients With Progressed and Stable Lesions

No significant differences were observed in demographics, radiographic parameters, treatment plan, type and location of the initial lesion, and muscle FI between hips with and without a lesion progression (Table 6). Patients with progressed lesions demonstrated significantly more trochanteric pain (VAS, 4.6 vs 2.8) and decreased hip abductor function (Trendelenburg positive in 4 [19%] vs 1 [2%]) than patients with a stable lesion (*P* = .0001) at the latest follow-up.

**Table 6:** Patient Demographics, Radiologic Parameters, Treatment, Type and Location of Initial Lesion, and Functional Status Between Patients With and Without Lesion Progression at Last Follow-up^*a*^

	Mean ± SD (Range) or No. (%)		
Characteristic	Progression (n = 21)	No Progression (n = 43)	*P* Value^*b*^
Demographics			
Age, y	70 ± 15 (33-91)	66 ± 14 (37-82)	.3
Female sex	11 (52)	33 (77)	.6
Right hip	8 (38)	28 (65)	.1
BMI	27 ± 5 (19-36)	27 ± 5 (19-36)	.9
Smoking	6 (29)	22 (51)	.2
Radiologic parameters			
Femoral offset, mm	49.8 ± 6.7 (37-63)	49.8 ± 6.7 (36-66)	.9
CCD angle,^*c*^ deg	129 ± 6 (115-140)	128 ± 6 (129-167)	.8
Osteoarthritis	13 (62)	22 (51)	.5
Treatment			
Physical therapy	10 (48)	26 (60)	.7
Infiltration cortisone	10 (48)	24 (56)	.8
Type of hip abductor lesion^*d*^			
Trochanteric bursitis	8 (38)	7 (17)	.2
Tendinopathy	6 (29)	8 (19)	.4
Partial rupture	5 (24)	26 (60)	.8
Scar	0	1 (2)	
Complete rupture	2 (9)	1 (2)	.7
Trochanteric facet initially involved^*e*^			
Anterior	11/13 (85)	31/36 (86)	.9
Lateral	11/13 (85)	32/36 (88)	.9
Posterior	3/13 (23)	17/36 (47)	.6
Muscle fatty infiltration : Goutallier/Fuchs^13^			
G*min*^*f*^	2 [1-3]	2 [1-3]	.8
G*med*^*f*^	1 [1-1.75]	1 [1-1]	.8
Progression of fatty degeneration	10 (48)	19 (44)	.7
Functional status			
HHS,^*c*,*e*^ points	75 ± 17 (30-99)	82 ± 13 (56-100)	.7
WOMAC,^*c*^ points	3.1 ± 2.3 (0-6.9)	2.3 ± 1.7 (0-6.9)	.7
VAS, trochanteric pain	4.6 ± 2.8 (0-10)	2.8 ± 2.6 (0-7)	**.001**
Trendelenburg	4 (19)	1 (2)	**.001**

a BMI, body mass index; CCD, centrum-collum-diaphyseal; G*med*, gluteus medius; G*min*, gluteus minimus; HHS, Harris Hip Score; VAS, visual analog scale; WOMAC, Western Ontario and McMaster Universities Osteoarthritis Index.

b Bold indicates significance (*P* < .05).

c As measured at the final follow-up.

d The most severe lesion was described.

e Patients with trochanteric bursitis but intact tendons were excluded.

f Median [interquartile range].

Multiple logistic regression analysis demonstrated no significant correlation among patient demographics (age, BMI, smoking status), radiologic parameters (CCD angle, femoral offset, osteoarthritis grade), type and location of the initial lesion, and lesion progression.

## Discussion

The best treatment of a hip abductor tendon lesion, a common and usually disabling cause of GTPS, remains largely unknown. Surgical intervention is usually recommended for refractory symptoms after 3 months of failed nonoperative treatment.^1,18^ As a result, the natural course of nonoperatively treated hip abductor lesions has never been reported. The purpose of the present study was to report the natural history of nonoperatively treated degenerative hip abductor lesions. At an average 6-year follow-up, one-third of nonoperatively treated hip abductor lesions demonstrated a progression on MRI. Although patients with a progressed lesion experienced more trochanteric pain, the functional outcomes were comparable with patients with a stable lesion. Interestingly, the majority (90%) of partial lesions remained stable. More than two-thirds of hips with a partial tear had a progression of G*min* FI, which differed significantly from the contralateral side, but only 1 progressed to high-grade FI. Despite the fact that almost half of the patients demonstrated a progression of G*med* FI, this did not differ from the contralateral asymptomatic side. No risk factors for lesion progression were identified.

The majority of hip abductor lesions were reported in the anterolateral trochanteric facet in imaging, surgical, and pathologic studies.^6,16,39^ Although hip abductor lesions involving the posterior facet are relatively rare, they tend to be more symptomatic.^29^ In accordance with the literature, the majority of degenerative hip abductor partial tears in the present study were observed in the anterior or lateral facet (60%), with the posterior facet being involved in just 4% of the partial tears. The pattern of hip abductor lesions suggests that degenerative abductor lesions might originate from the anterolateral facet, as the posterior facet is rarely involved in the degenerative process. Functionally, the G*min* acts synergistically with the anterior G*med* to stabilize the pelvis during gait, with several electromyographic studies reporting increased loads in these muscles during the single-stance phase of gait, which might predispose to tendinopathy and tear.^33,34^

Surgical repair of hip abductor tears demonstrated satisfactory outcomes with the HHS, ranging from 61% to 88% for open^7,10^ and 74% to 94% for arthroscopic techniques, with relatively low retear rates (7% for arthroscopic vs 6%-25% for open techniques).^3,5,8,38^ Specifically, Bogunovic et al^3^ reported an average HHS of 81 points and a VAS of 1.7 after arthroscopic repair of hip abductor lesions in 30 patients with an average follow-up of 35 months. Similar results were cited by Domb et al^8^ (HHS, 85 points; VAS, 1.4) in 15 patients at an average 28 months of follow-up and by Chandrasekaran et al^5^ (VAS, 2.4) in 34 patients at an average 27 months of follow-up. In patients with severe pain (VAS >6), endoscopic hip abductor tendon repair could significantly lower their pain scores—from 6.6 to 2.4 and from 6.2 to 2.6 (*P* = .001) in 2 studies^5,8^—and improve their function by eliminating their Trendelenburg sign in a majority of the cases.^8^ Similar functional outcomes have been noted with open techniques, with Fearon et al^10^ reporting an average HHS of 71 points in 18 patients with an average follow-up of 22 months. In accordance, Davies et al^7^ cited an average HHS of 88 points in 23 patients at a mean 71 months of follow-up. Although a direct comparison with endoscopic hip abductor repair cannot be performed, as different hip outcomes scores were used (modified HHS and VAS, as well as the Hip Outcome Score with Activities of Daily Living and Sport-Specific subscales), the present study reported an average HHS of 80 points and VAS score of 3.4 after nonoperative treatment of hip abductor lesions. Although the HHS was worse than the contralateral/nonaffected side, the outcomes were similar to those in the literature for arthroscopic or open tendon repair.

Even though early surgical repair is commonly recommended in patients with hip abductor tears to avoid FI of the muscles,^17^ no data exist on the muscle FI after hip abductor tendon repair in native hips, and there is limited evidence regarding the effect of muscle FI on the surgical outcome. In a retrospective study of 30 hips treated with endoscopic hip abductor repair, Bogunovic et al^3^ reported that increased preoperative FI correlated with increased postoperative pain levels and decreased patient-reported outcomes and patient satisfaction. Contrarily, in a consecutive series of 84 patients with partial and full-thickness tears of the G*min*, with the anterior portion of the G*med* treated with open repair augmented with a LARS ligament, Ebert et al^9^ reported that muscle FI was not associated with patient outcomes, including pain, symptoms, functional capacity, perceived improvement, and satisfaction, and they concluded that surgical repair might be considered even in the presence of severe FI. In the present study, more than two-thirds of the G*min* FI in hips with partial tear progressed from grade 1 to 2, whereas just 1 hip (3%) progressed from grade 2 to 3 at the last follow-up. Only 3 hips (9%) demonstrated a progression of G*med* FI in hips with a partial tear, but no significant difference was observed as compared with the contralateral side, implying that it might be due to the natural muscle degeneration occurring with increasing age, as observed in the rotator cuff^15^ and paraspinal muscles.^27^ A recent meta-analysis of 206 hips found that high-grade FI (grades 3 and 4) was significantly associated with less improvement in HHS than no FI or low-grade FI (grades 1 and 2) but did not influence the VAS.^24^ These results suggest that partial tears of the hip abductors should not be considered an absolute indication of early surgical repair, as clinically relevant FI was not observed.

The present study should be interpreted in light of its potential limitations. The most obvious challenge was to report the size of the tendon rupture (length and width). As degenerative hip abductor ruptures typically occur as tendon delamination involving the undersurface of the tendon without tendon retraction,^39^ the tear size could not be adequately assessed in MRI scans. Nevertheless, the method used to describe the lesions in the present study allowed investigation of lesion progression. Another drawback was that the average time between initial and final MRI examination was 6 years, and no serial MRI scans were performed in the meantime. Consequently, the dynamics of lesion progression (sudden or gradual) could not be assessed. However, sequential MRI scans were not performed in the present study because of the high cost of MRI and relatively low clinical relevance. Furthermore, a control group with operatively treated hip abductor lesions was not available for a direct comparison. Instead, the results of the present study were compared with the functional outcomes of hip abductor lesions treated arthroscopically or with an open repair, as reported in the literature. Moreover, 48 of 106 (45%) patients declined to participate, so it is not known if the results reflect the natural history of these patients. Also, in the present study, only 3 patients with complete tears at enrollment were included; therefore, the natural history of complete tears demonstrated should be interpreted with caution. Additionally, it should be emphasized that the WOMAC and HHS do not assess sports activity, so this aspect of function could not be assessed. Finally, the VAS for pain and clinical examination were not assessed at enrollment; as such, a possible progression of pain or increase in Trendelenburg limp could not be assessed.

In conclusion, to the best of our knowledge, the current study is the only one available in the literature reporting the natural history and structural outcome (lesion progression of FI) of degenerative hip abductor lesions on sequential MRI scans. About one-third of hip abductor lesions progressed over time, with patients with lesion progression demonstrating significantly more trochanteric pain (VAS, 4.6 vs 2.8) and decreased hip abductor function (Trendelenburg positive in 19% vs 2%) than patients with a stable lesion. Because recent studies showed that endoscopic hip abductor repair could significantly reduce pain and function in patients with severe pain (VAS >6),^5,8^ nonoperative treatment might not be the best approach in patients who have severe trochanteric pain and degenerative changes shown on MRI. Furthermore, the majority (90%) of partial lesions remained stable, whereas just 1 complete tear progressed to another trochanteric facet. Despite the fact that almost half of the patients had a progression of G*med* FI, this did not differ from the contralateral/unaffected side, implying that this finding might be due to the natural muscle degeneration occurring with increasing age. Contrarily, more than two-thirds of hips with a partial tear demonstrated a progression of G*min* FI, which differed significantly from the contralateral side, but only 1 progressed to high-grade FI. In light of these findings, it appears that nonoperative treatment might be a valid long-term option for degenerative hip abductor lesions, especially for partial tears that demonstrated a low risk of clinically relevant progression or muscle FI and similar clinical outcomes as reported in the literature for the operatively treated hip abductor tendon lesions. Further prospective randomized controlled trials with serial MRI scans are warranted to investigate the outcomes of nonoperative and operative treatment in patients with degenerative hip abductor tendon lesions.
